# Wrapping of the Gastroduodenal Artery Stump to Reduce Haemorrhage After Pancreaticoduodenectomy

**DOI:** 10.7759/cureus.95739

**Published:** 2025-10-30

**Authors:** Sulaiman Hussain, Anse Arif, Haroon Zaffar, Haris Shoaib, Mooyad Ahmed, Petru Bucur, Mohamed Bekheit

**Affiliations:** 1 Department of Surgery, Royal Blackburn Teaching Hospital, Blackburn, GBR; 2 Department of Surgery, University of Central Lancashire, Preston, GBR; 3 Department of Trauma and Orthopaedics, Royal Bolton Hospital, Bolton, GBR; 4 Department of Trauma and Orthopaedics, Royal Preston Hospital, Preston, GBR; 5 Department of Surgery, Regional University Hospital Centre (CHRU) of Tours, Tours, FRA; 6 Department of Surgery, University of Aberdeen, Aberdeen, GBR

**Keywords:** gastroduodenal artery stump, gda wrapping, pancreaticoduodenectomy, post-pancreatectomy haemorrhage, whipple's procedure

## Abstract

Pancreaticoduodenectomy (PD) is a complex and high-risk surgical procedure associated with significant morbidity and mortality, particularly due to post-pancreatectomy haemorrhage (PPH). Wrapping the gastroduodenal artery stump (GDAS) intraoperatively has been proposed as a preventive measure, utilizing various materials such as the falciform ligament (FL), ligamentum teres hepatis (LTH), omentum, and polyglycolic acid (PGA) sheets. A comprehensive meta-analysis was conducted, including 10 studies involving 3,398 participants. Comparative studies were selected to evaluate the risk of PPH with GDAS wrapping versus control groups. Odds ratios (ORs) were used to assess efficacy. GDAS wrapping significantly reduced PPH incidence (OR = 0.23, 95% CI: 0.11-0.51), despite high heterogeneity among studies (I² = 64%). Subgroup analysis showed significant reductions with FL/LTH (OR = 0.20, p = 0.003) and PGA (OR = 0.18, p = 0.007) but not with omental (OR = 0.37, p = 0.07). The analysis of 30-day mortality did not reach significance (OR = 0.76, p = 0.44). GDAS wrapping may reduce PPH risk; however, further randomised studies are needed to confirm these findings and assess the superiority of specific wrapping techniques.

## Introduction and background

Pancreaticoduodenectomy (PD) is a complex and high-risk procedure, primarily performed for tumours of the pancreatic head, periampullary region, duodenum, and distal common bile duct, albeit for non-cancer pathologies [1]. It is associated with high rates of perioperative morbidity and mortality with complication rates reported between 30% and 50% [2,3]. Common post-operative complications following PD include delayed gastric emptying (15%-25%), post-operative pancreatic fistula (POPF) (10%-30%), intra-abdominal abscesses, biliary leakage, and post-pancreatectomy haemorrhage (PPH) [4].

Among the most serious complications following PD is post-pancreatectomy haemorrhage (PPH), occurring in approximately 5%-10% of patients [5]. Despite its moderate incidence, it accounts for the greatest morbidity and mortality burden by significantly impacting hospital stay and patient recovery [5]. Recent evidence has quantified this in population attributable fractions (PAFs) of 32.8% for mortality and 22.1% for organ failure [6]. The severity of post-pancreatectomy haemorrhage has been defined by the International Study Group of Pancreatic Surgery (ISGPS), where time course, location, and clinical features equate to a Grade A, B, or C classification [4]. Condition severity escalates across the classification, and this is reflected in the mortality rates, with Grades A, B, and C reported at 4.2%, 3.9%, and 28.5%, respectively [7].

This post-operative bleeding has been most localised to the gastroduodenal artery stump (GDAS). Aetiologies for PPH include processes such as vascular erosion secondary to pancreatic leakage and from post-operative pancreatic fistula or dehiscence of the anastomosis [8]. CT angiographic evidence localised the source of PPH to the GDAS in 29% of cases; this was followed by the common hepatic artery (19%) and splenic artery (12%) [5]. This GDAS bleeding has been shown to result in both intraluminal and extraluminal bleeding [5]. Clinical features such as haematemesis and per rectum bleeding are suggestive of intraluminal bleeding, whereas haemodynamic instability and fresh blood draining from the abdomen are indicative of extraluminal bleeds [7]. As such, the GDAS has become a focal area in prevention and intervention of PPH.

Intraoperative approaches for vascular protection can vary [9]. These approaches include the use of wrapping techniques with biological or synthetic materials such as the falciform ligament (FL), ligamentum teres hepatis (LTH), omentum, and polyglycolic acid (PGA) sheets to reduce the incidence of PPH [10,11]. This wrapping provides reinforcement to the GDAS, or more proximally at the hepatic artery, with the aim of isolating this arterial plexus from potential pancreatic enzymes, reducing the incidence of bleeding, and bleeding-associated complications. This article and meta-analysis builds upon previous literature by providing an updated analysis of the available literature. This article also evaluates subgroup differences and provides additional insight into mortality outcomes, aiming to clarify the potential role and effectiveness of each wrapping technique, as we hypothesise that wrapping of the GDA can effectively reduce rates of haemorrhage following PD.

## Review

Methods

Study Selection

The study follows the Population (or patient/problem), Intervention (or exposure), Comparator (or control), Outcome(s), Study design (PICOS) structure. All included patients must have undergone PD. Patients in the intervention cohorts must have undergone wrapping of the GDA, whether in a primary intent or through wrapping of the hepatic artery. All surgical techniques achieving this were included. Interventions aside from wrapping in this location were excluded. Only comparative studies evaluating the risk of PPH between cohorts with and without GDA wrapping following PD were included. Non-comparative studies or studies failing to provide event data were excluded. Further to this, a patient's disease status and indication for surgery was not considered in study selection; there was no restriction on patient age or gender. Studies were selected if they reported this review’s primary outcome in their primary or secondary outcome measures. Only English-language articles were considered.

Outcome Measure

The primary outcome measure was the incidence of PPH as defined by ISGPS, with all grades A-C considered. Moreover, both early and late haemorrhages, as well as both intra- and extraluminal haemorrhages, as potential manifestations of GDAS bleeds, were included in the measure [12]. The secondary outcome analysed was 30-day mortality. Measures of effect for all outcome measures was described through odds ratios (ORs) across study arms. In this instance, hazard ratios were not used as the studies included did not provide sufficient time-to-event data. These data points would be necessary when inferring survival analysis.

Search Methods for Identification of Studies

In October 2023, electronic searches of papers in the English language were conducted using the following databases: Medline, Embase, CENTRAL (Cochrane), and PubMed. The following search terms were used: ((Whipple OR pancreaticoduodenectomy)) AND ((Wrap* OR Cover* OR Floor* OR Ligament)) AND ((gastroduodenal artery OR GDA or major vessels OR haemorrhage). These articles were subsequently screened for relevance, using Rayyan.AI online [13] by three independent reviewers. Once all three reviewers had screened all articles, the results of the screening process were unblinded, and any disputes were discussed, until resolved, and a consensus was achieved. The bibliographies of included articles were subsequently screened for relevant articles. After deduplication, these were then screened through the same process as listed above.

Data Extraction and Management

The PICOS structure was used to template the data extraction [14] Two initial reviewers (SH, AA) independently extracted data into an Excel (Microsoft Corporation, Redmond, Washington, United States) spreadsheet, with a third (NR) checking the reviewed data. Disagreements on extracted data were resolved via discussion. Following this, data was input into RevMan Review Manager 5.4 (The Cochrane Collaboration, Copenhagen, Denmark) for analysis [15,16]. Study investigators were to be contacted for additional details on missing data if this arose.

Data Collection and Analysis

RevMan 5.4 was used to synthesise the data. Data is presented using OR with 95% confidence intervals (CIs) [16]. The Mantel-Haenszel methods were used in this meta-analysis. Sum effect is illustrated with a forest plot. Heterogeneity is represented via an I² value. Random effects model was implemented. Subgroup analyses were performed for each type of wrapping material (FL/LTH, omental, PGA). Subgroups including only a single study (e.g. PGA) were interpreted as hypothesis-generating rather than confirmatory.

Assessment of Risk of Bias in Included Studies

The Risk Of Bias In Non-randomized Studies of Interventions (ROBINS-I) tool was used to assess risk of bias of included non-randomised studies [15,17]. Confounding factors assessed for were male sex, BMI > 25, serum Alb < 3.5 g/l, and presence of diabetes mellitus (DM) (glycated haemoglobin A1c (HbA1c) level > 6.2%). Any co-interventions were identified and assessed as per the ROBINS-I protocol. For randomised studies included in this review, the Risk of Bias 2 (RoB2) tool was used [16].

Limitations

The studies included measured mortality and post-operative pancreatic leak as secondary outcomes; however, there were discrepancies to the definition to these outcomes. For this review’s secondary outcome, two studies defined mortality as 90-day mortality, and one study defined this outcome as 60-day mortality. One study did not specify the period for the definition of mortality [18-21].

Results

Results of the Search and the Quality of the Evidence

**Figure 1:**
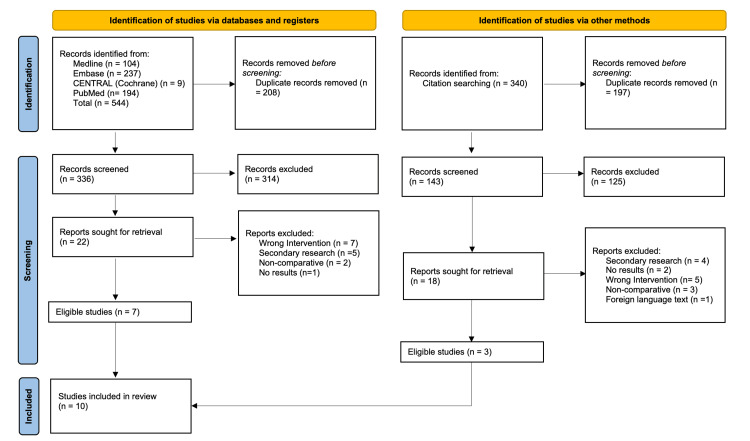
PRISMA flowchart outlining study selection From Page et al. [17]. PRISMA: Preferred Reporting Items for Systematic reviews and Meta-Analyses.

One randomised controlled trial (RCT) and nine retrospective studies on 3,398 participants were included in the meta-analysis [22-33]. The process of inclusion of the trials is detailed in the Preferred Reporting Items for Systematic reviews and Meta-Analyses (PRISMA) chart (Figure 1) [17]. The included trials were conducted in China, Germany, Japan, Greece, India, and Korea. All the studies were performed between 2007 and 2023. There was no discrimination for study selection in terms of gender, age, and number of recruited patients. A summary of the characteristics of included studies is detailed below (Table 1).

**Table 1:** Summary of characteristics for included studies PD: pancreaticoduodenectomy, PGA: polyglycolic acid sheet, LTH: ligamentum teres hepatitis, PPH: post-pancreatectomy haemorrhage; POPF: post-operative pancreatic fistula; DGE: delayed gastric emptying, FL: falciform ligament.

Study	Design	Participant s	Intervention	Control	Outcomes Measured
Kapoor et al. [23]	Single-centre retrospective cohort	n = 77	Omental flap n = 25	PD without omental flap n = 52	Intra-abdominal haemorrhage, anastomotic leak, 30-day mortality
Lee et al. [22]	Multicentre retrospective cohort	n = 904	PGA wrap n = 491	PD without PGA n = 413	PPH, post-operative complications, mortality
Meng et al. [24]	Single-centre retrospective cohort	n = 247	LTH wrap n = 119	PD without LTH wrap n = 128	PPH, POPF, intra-abdominal abscess, DGE, length of operating time, post-op hospital stay, interventions to treat outcomes
Mussle et al. [25]	Single-centre retrospective cohort	n = 196	FL wrap n = 39	PD without FL wrap n = 157	POPF, erosion haemorrhage, 60-day mortality
Okada et al. [26]	Single centre, retrospective cohort	n = 500	FL wrap n = 193	PD without FL n = 307	PPH, POPF, 90-day mortality
Shah et al. [27]	Single centre, retrospective cohort	n= 147	Omental wrap n = 101	PD without omental wrap n = 46	PPH, POPF, DGE, intra-abdominal abscess, 30-day mortality
Welsch et al. [29]	Multicentre randomised controlled trial	n = 445	FL wrap n = 222	PD without FL wrap n = 223	PPH, POPF, rate of hepatic malperfusion and stenosis of hepatic artery, reoperation rates, morbidity, and death
Xu et al. [30]	Single centre, retrospective cohort	n = 280	LTH wrap n= 140	PD without LTH wrap n= 140	PPH, POPF, intra-abdominal infection, DGE, mortality
You et al. [31]	Single centre, retrospective cohort	n = 454	FL wrap n = 326	PD without FL wrap n= 128	POPF, pancreatic leak, intra-abdominal erosive haemorrhage, 90-day re-operation, 90-day mortality
Yu et al. [32]	Single centre, retrospective cohort	n = 148	LTH wrap n= 85	LPD without wrapping n = 63	PPH, POPF, DGE, abdominal infection, post-operative hospital admission, death

Primary Outcome (Post-pancreatectomy Haemorrhage)

**Figure 2:**
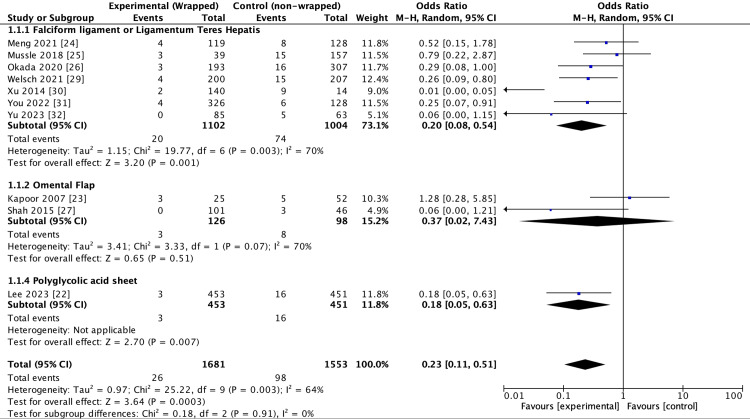
Forest plot of outcome: post-pancreatectomy haemorrhage From references [22-27,29-32].

The wrapping of GDA significantly reduced overall haemorrhagic complications in comparison to non-wrapping (OR = 0.23 (0.11-0.51, 95% confidence interval)). Additionally, a high degree of heterogeneity was observed between the included studies (I2 = 64%, p = 0.003) (Figure 2). Subgroup analysis was performed on the following three groups: FL/LTH (seven studies, n = 2,106), omental (two studies, n = 224), and PGA (one study, n = 904), to assess odds of PPH for each individual wrapping modality. At baseline, these groups exhibited, where applicable, low heterogeneity, which did not reach statistically significance (I2 =0%, p = 0.91). The use of FL/LTH and PGA sheet both demonstrated a reduction in incidence of PPH, with OR 0.20 (0.08-0.54, 95% CI, p = 0.003) and OR 0.18 (0.05-0.63, 95% CI, p = 0.007), respectively. However, the analysis of the odds of PPH when using the omental flap trended toward significant however failed to reach significance, OR 0.37 (0.02-7.43, 95% CI, p = 0.07) (Figure 2).

Secondary Outcome (Post-operative Mortality)

**Figure 3:**
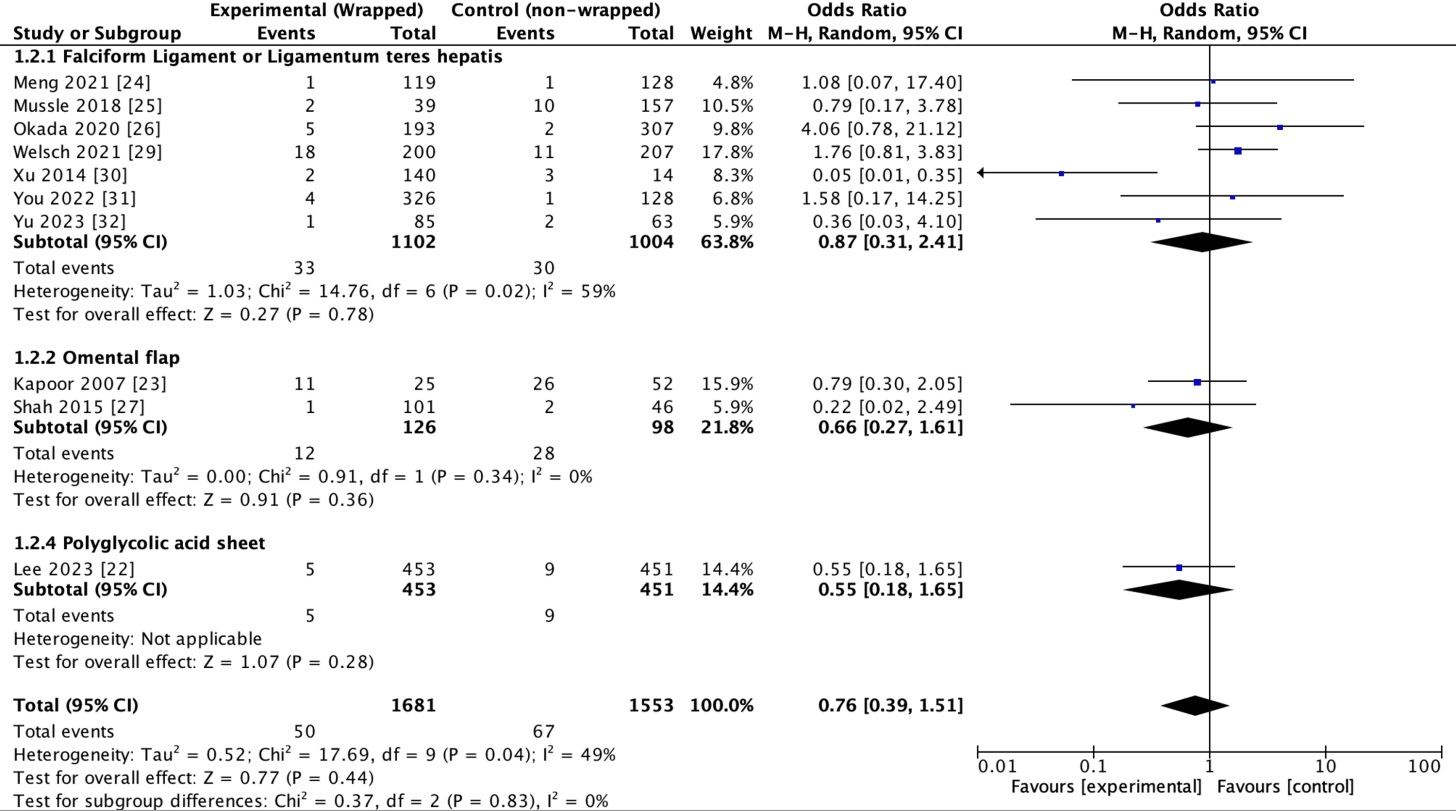
Forest plot outcome: 30-day mortality From references [22-27,29-32].

Analysis on odds of 30-day mortality between wrapping and control did not reach significance, OR 0.76 (0.39-1.51, 95% CI, p = 0.44). Notably, however, a moderate degree of heterogeneity was observed in the pool of included studies (I2 = 49%, p = 0.04) (Figure 3). The subgroup analysis was similarly conducted on the same three groups: FL/ LTH (seven studies, n = 2.106), omental (two studies, n = 224), and PGA (one study, n = 904). At baseline, there was low subgroup heterogeneity which did not reach significance (I2 = 0%, p = 0.83). Moreover, the analysis of odds of post-operative mortality did not reach significance in any of the subgroups: FL/LTH OR = 0.87 (0.31-2.41, 95% CI, p = 0.78), omental OR = 0.66 (0.27-1.61, 95% CI, p = 0.36), and PGA OR = 0.55 (0.18-1.65, 95% CI, p = 0.28) (Figure 3).

Bias Assessment

**Figure 4:**
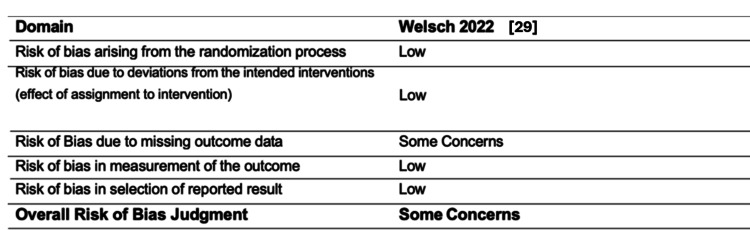
RoB2 risk of bias assessment for randomised studies From Sterne et al. [16] and Welsch et al. [29]. RoB2: Risk of Bias 2.

The risk of bias assessment for non-randomised studies (Table 2) was conducted using the ROBINS-I tool. Of the nine non-randomised studies, one was deemed moderate in risk of bias, five severe, one critical, one moderate/not classified, and one was unclassified. Randomised studies were assessed using the RoB2 tool (Figure 4), with the single included study showing some concerns.

**Table 2:** ROBINS-I risk of bias assessment for non-randomised studies From Sterne et al. [15]. ROBINS-I: Risk Of Bias In Non-randomized Studies of Interventions.

Domain	Shah et al. [27]	Okada et al.[26]	Kapoor et al. [23]	Meng et al. [24]	Xu et al. [30]	You et al. [31]	Yu et al.[32]	Lee et al. [22]	Müssle et al. [25]
Bias due to confounding	Moderate risk	Serious risk	Serious risk	Moderate risk	Serious risk	Serious risk	Serious risk	No risk	Serious risk
Bias in selection of participants into the study	Low risk	Low	No information	Low	Low	No information	Low	No information	Critical risk of bias
Bias in classification of interventions	Low risk	Low	Low	Low	Low	Low	Low	Low	Low
Bias due to deviations from intended intervention	Low risk	Low	Low	No information	Low	Serious	Low	No information	Low
Bias due to missing data	Low risk	Low	Low	Low	Low	No information	No information	No information	No information
Bias in measurement of outcomes	Low risk	Moderate	Low	Low	Low	Low	Low	No information	Low risk
Bias in selection of the reported result	Moderate	Moderate	Moderate	Moderate	Moderate	No information	Moderate	No information	Moderate risk of bias
Overall bias judgment	Moderate	Serious	Serious	Moderate/Low	Serious	Serious	Serious	No information	Critical risk

Discussion

PPH is a clinically significant event, with profound impact on patient morbidity and mortality. The results of this meta-analysis, derived from an analysis of a total of 3,398 patients across 10 studies, suggest that wrapping the GDAS significantly reduces the incidence of PPH, when compared to controls. However, 30-day mortality was not significantly different between groups (OR = 0.76, 0.39-1.51, 95% CI). This study, being the only investigation of this parameter with this size of cohort using a rigorous approach, reinforced the principle of GDAS wrapping benefiting PPH reduction rather than overall post-operative mortality. A prior systematic review performed by Nour et al. 2022 highlighted the lack of prospective and randomised controlled studies assessing GDAS wrapping [9]. Although randomised controlled studies on this topic have started to emerge in recent years, the majority of literature remains retrospective and non-randomised in nature.

Compared with the systematic review by Nour et al. (2022) [9], which included two randomised controlled trials (RCTs) and a similar patient population, our study presents novel contributions by incorporating more recent data, including cohort studies and the first meta-analysis evaluating synthetic wrapping materials such as polyglycolic acid (PGA) sheets. This material-specific subgroup analysis has not been explored in this context previously and offers an opportunity to compare effectiveness among the various materials [9]. It is also important to note the impact of the sample size included in our review and its weight in adding further legitimacy to the statistical conclusions made as a result of the analysis.

The derived results provide evidence in favour of GDAS wrapping; however, the heterogeneity amongst the pooled studies must be considered when interpreting the external validity of these findings, as well as the fact that only one study was included in this subgroup. This is consistent with previous pooled findings favouring the wrapping [9]. However, this analysis found high heterogeneity across the pooled studies (I2 = 64%), which is greater than has been observed in previous review (I2 = 36%) [9]. This could be attributed to the broader study selection, with the inclusion of 10 studies, compared to the seven studies included in the previous review [9]. The variation in wrapping methods may also account for the marked heterogeneity, which reached significance in both the primary and secondary endpoint. Furthermore, there are limitations in study quality, with six of the included studies having at least a serious risk of bias (Table 2). The susceptibility of non-randomised studies to systematic bias may also have contributed to observed heterogeneity.

The findings derived from our analysis are in alignment with the previous studies as stated; however, the inclusion of newer studies in our analysis allows for a more targeted approach when investigating each of these methods, and as such, this study serves its purpose as being an impactful addition to this field’s evidence base. In addition to this, the high heterogeneity observed across all included studies (I2 = 64%) underlines the breadth of the inclusion of our study and subsequent variability, which has its benefit in preventing interpretation of tightly controlled cohorts.

The included wrapping materials were diverse in origin and characteristics. The falciform ligament has previously been utilised in a variety of intra-abdominal surgery, with its utility attributed to it being both fibrous and vascularised, whilst being accessible in hepatobiliary procedures [22,23]. Conversely, the omentum is a fenestrated membranous organ, which is highly vascularised and offers angiogenic, haemostatic, and regenerative properties [24,25]. It has been demonstrated to be effective in adherence and as a sealant and offers a potential solution to prevent anastomotic leak or haemorrhage [25]. PGA, through its synthetic nature, offers a reproducibility and consistency, that the anatomical variation of the other two materials is unable to ensure. Furthermore, PGA might spare the patient of associated complications from mobilization of the falciform ligament and omentoplasty, albeit rare [26]. This added context into the practical aspects of each of these wrapping methods indicate the need for further investigation between each method in a more comparative manner.

Power calculations revealed that, for a powered effect size of 80% on the primary outcome and at 5% level of significance, an estimated n = 1,861 is the minimum required to reliably demonstrate a significant difference in the odds of PPH between wrapping and controls. The main analysis of this review demonstrates a sample size exceeding the threshold for reliable effect size. One subgroup analysis exceeded this threshold, FL/LTH (n = 2,106), with two subgroups falling short of this required sample size, omental (n = 224) and PGA (n = 904).

Discussion of Limitations

Due to lack of availability of patient-level data required to control for pancreatic leaks as a confounding factor for the PPH, this analysis was not possible. Pancreatic fistula is a significant cause of PPHs, further analysis to adjust for confounders would mitigate this burden, better isolating the effect size of GDAS wrapping. In future studies, the investigation of this parameter could be improved by the consistent reporting of the severity of POPF using standardised measures such as the ISGPS classification, allowing for further analysis of this outcome using a multivariate approach rather than a binary one.

Further limitations include the inconsistent definitions of mortality and POPF across the multiple studies, with different follow-up periods and grading systems. The subgroup analysis of the PGA group was based on a single study, thereby requiring cautious interpretation. This outlines an important absence in the current available literature, the need to assess synthetic wrapping materials with the same scrutiny as that which has been done with the biological methods. This study has identified this gap that future work should target.

In summation, several recommendations can be made for future much-needed work on this topic. Firstly, there is a need for distinct and standardised definitions and criteria for PPH, POPF, and mortality across all included studies. In addition, the incorporation of pancreatic fistulae and other confounders into the analysis will allow for a more meaningful conclusion to be made. As stated, the need for more powered studies, such as randomised controlled trials on these topics, will allow for a more statistically significant conclusion to be made regarding the importance of GDAS wrapping and specifically which methods have the impact observed.

## Conclusions

This study’s findings support that the use of wrapping of the GDAS may reduce the risk of post-pancreatectomy haemorrhage. Techniques such as the FL/ LTH have demonstrated a significant beneficial treatment effect. PGA sheet wrap may have advantages, but currently, only a single study was included. This review included multiple quality studies; however, future sufficiently powered studies and ultimately level 1 evidence, controlling for the occurrence of pancreatic leak, are required before firm conclusions can be drawn about the treatment effect of GDAS wrapping and furthermore of specific technique superiority.
